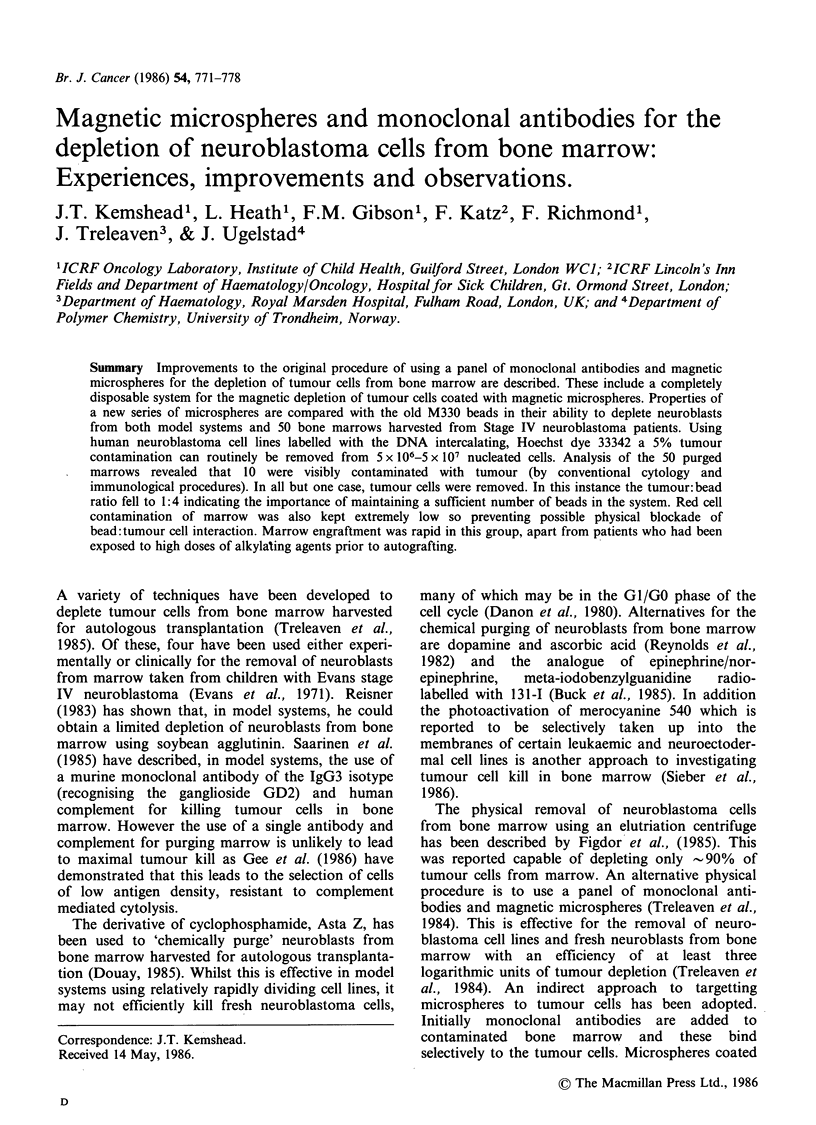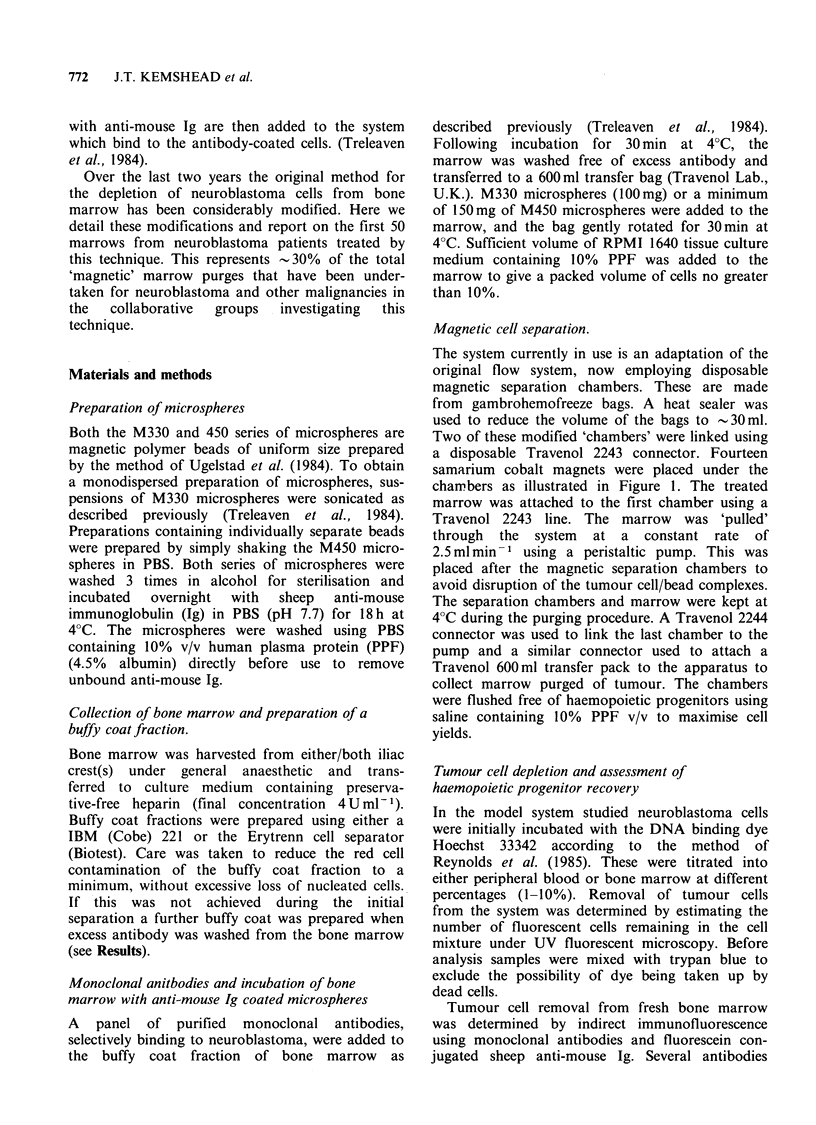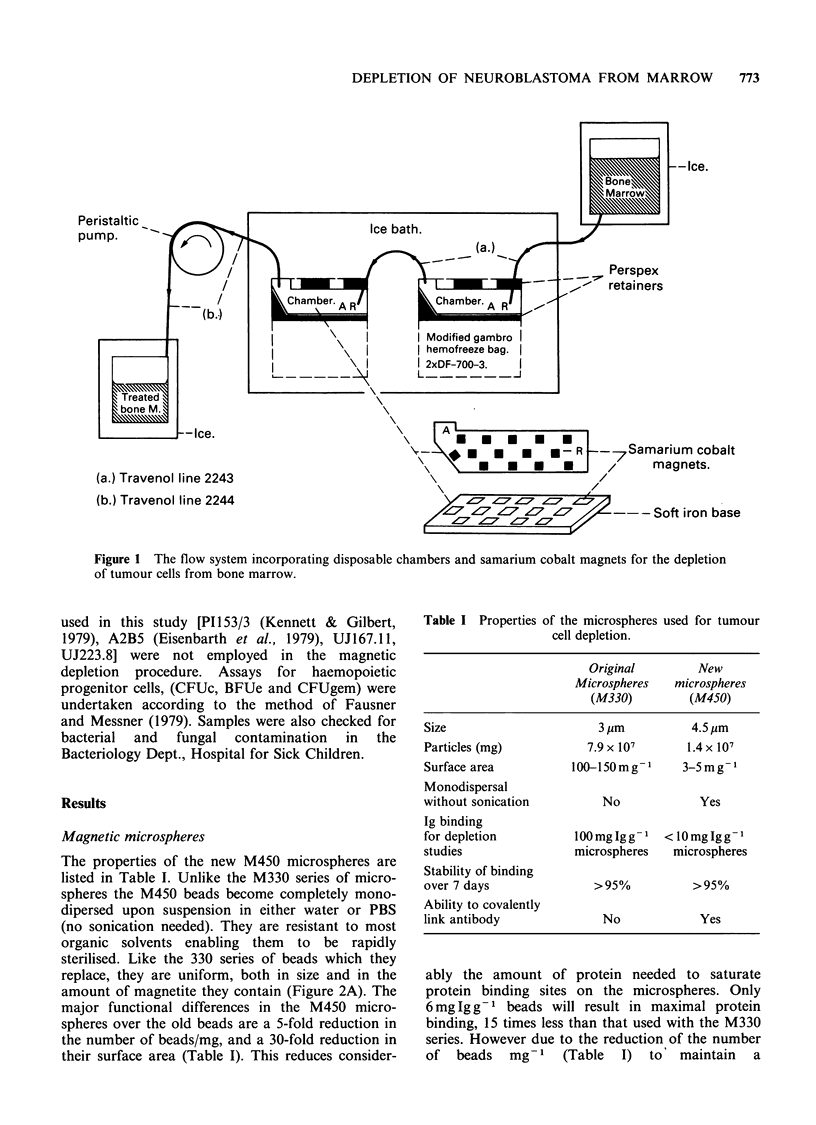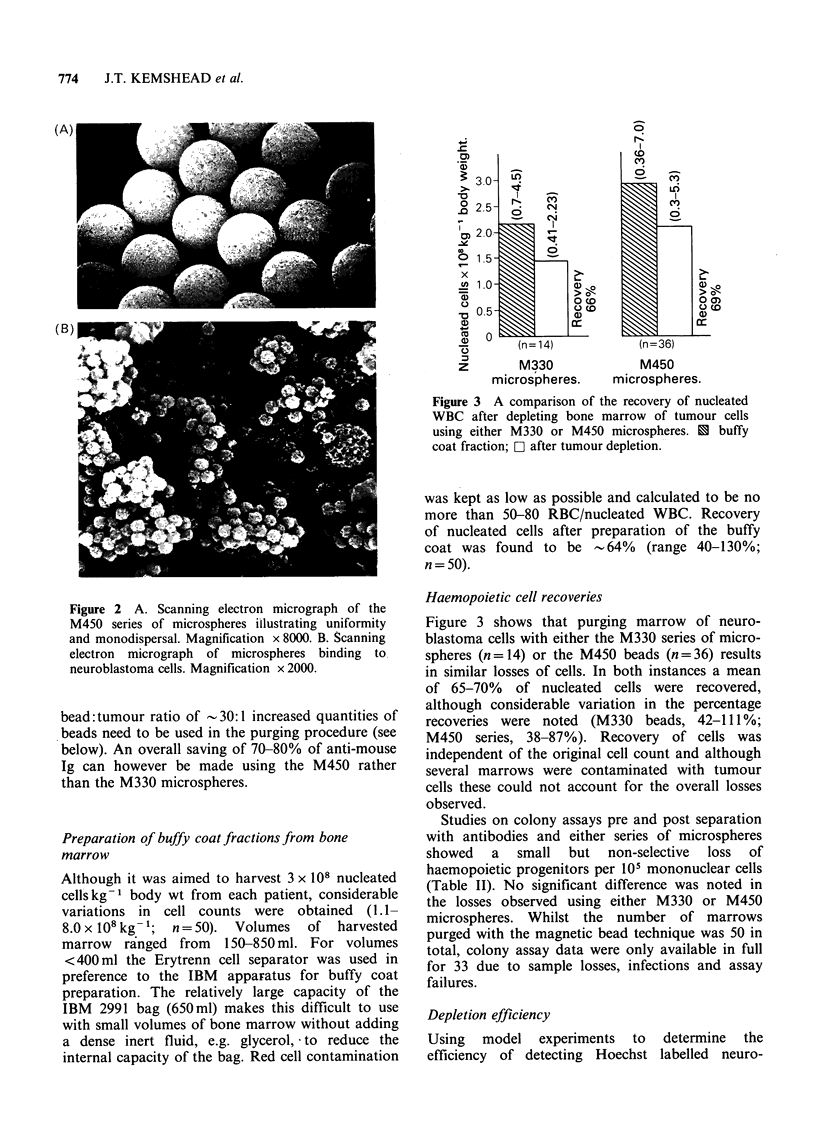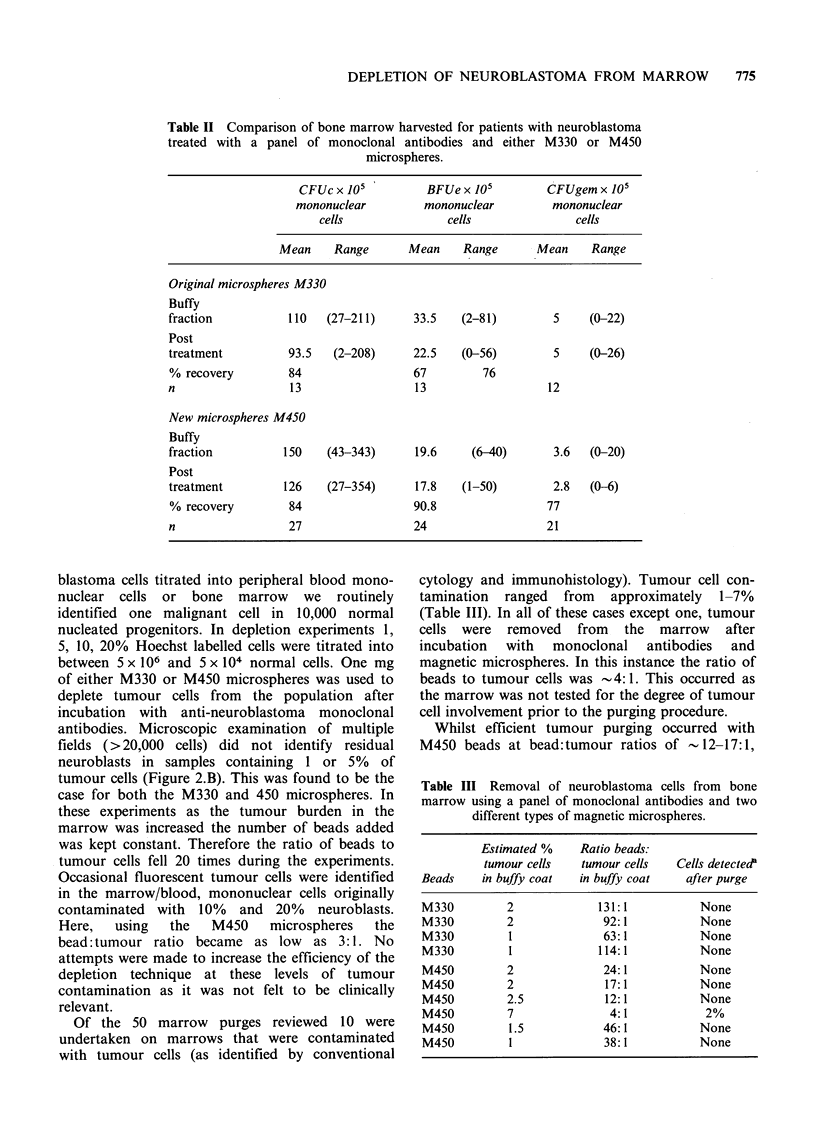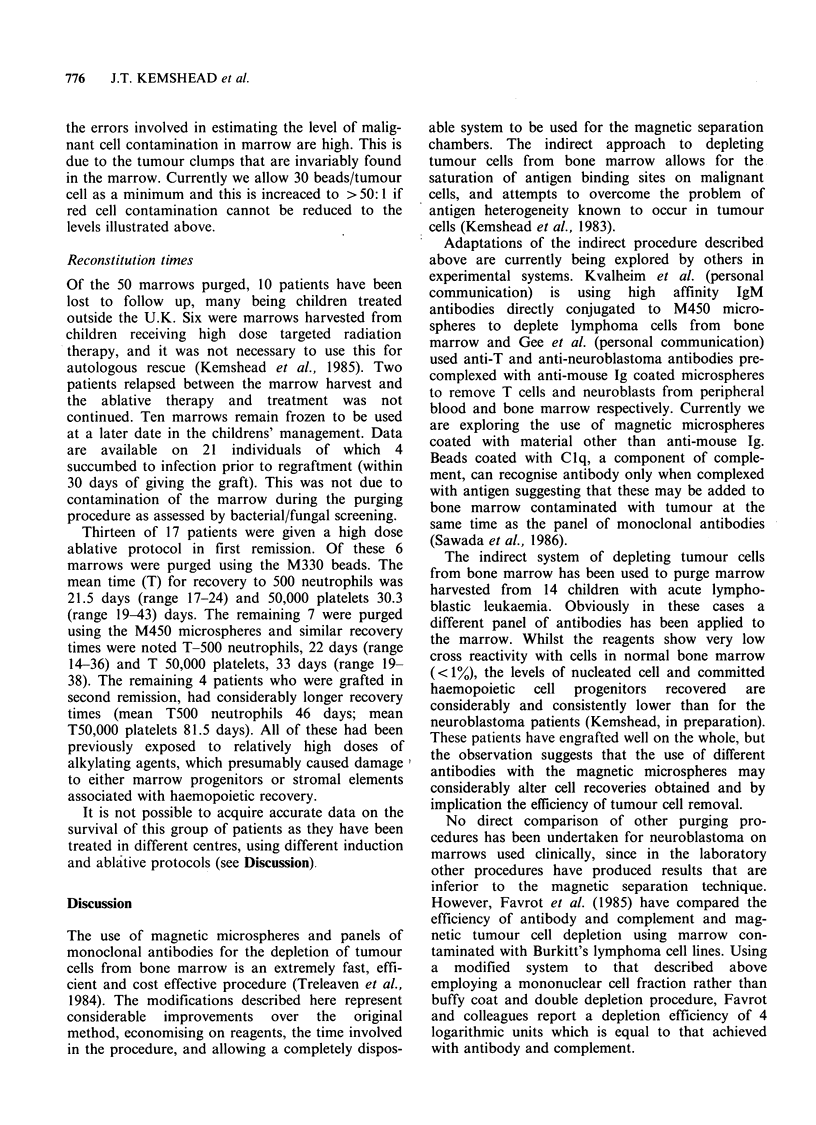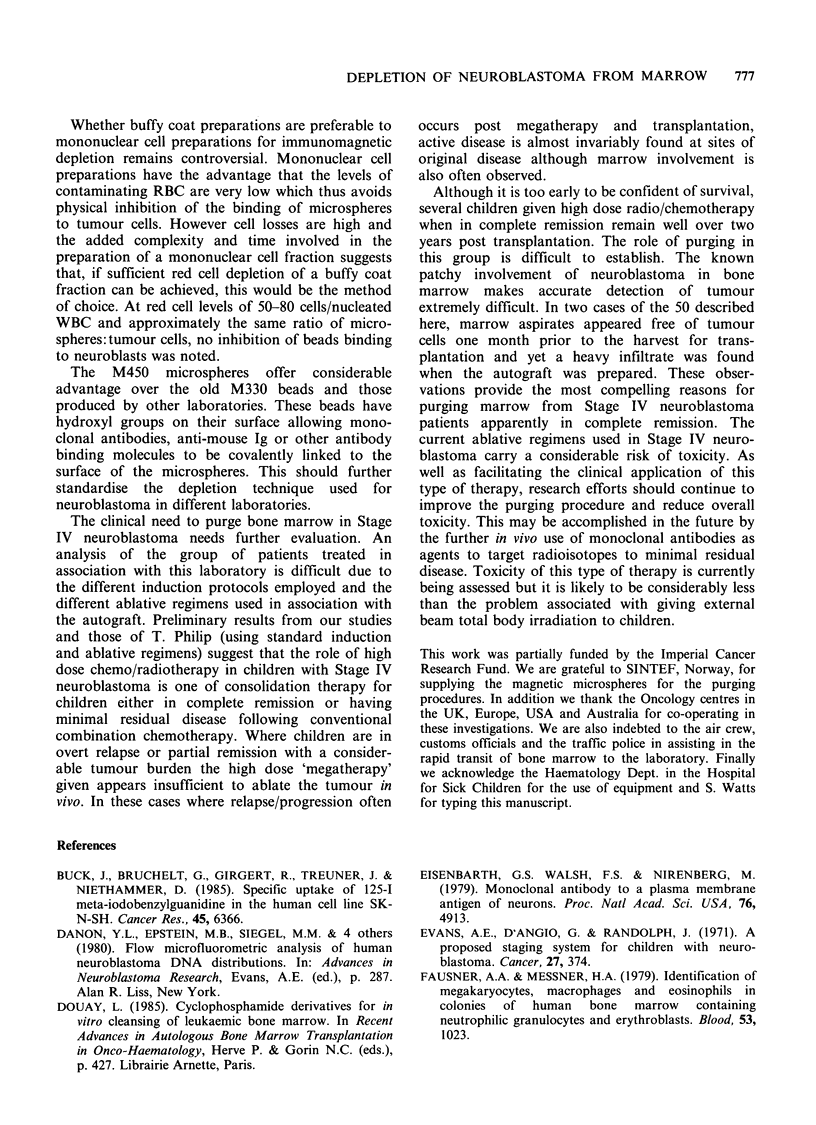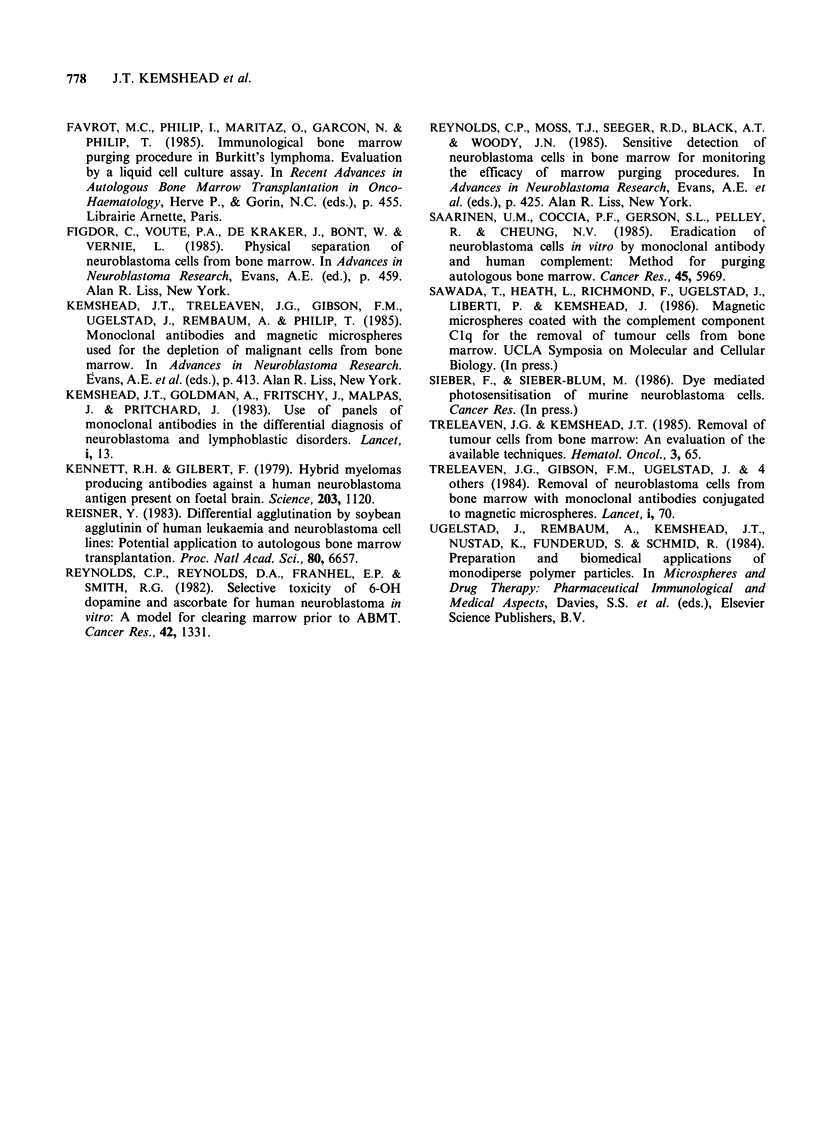# Magnetic microspheres and monoclonal antibodies for the depletion of neuroblastoma cells from bone marrow: experiences, improvements and observations.

**DOI:** 10.1038/bjc.1986.239

**Published:** 1986-11

**Authors:** J. T. Kemshead, L. Heath, F. M. Gibson, F. Katz, F. Richmond, J. Treleaven, J. Ugelstad

## Abstract

**Images:**


*Br. J. Cancer* (1986) **54**, 771–778

### Magnetic microspheres and monoclonal antibodies for the depletion of neuroblastoma cells from bone marrow: Experiences, improvements and observations.

J.T. Kemshead[1], L. Heath[1], F.M. Gibson[1], F. Katz[2], F. Richmond[1], J. Treleaven[3], & J. Ugelstad[4]

[1]*ICRF Oncology Laboratory, Institute of Child Health, Guilford Street, London WC1;* [2]*ICRF Lincoln's Inn Fields and Department of Haematology/Oncology, Hospital for Sick Children, Gt. Ormond Street, London;* [3]*Department of Haematology, Royal Marsden Hospital, Fulham Road, London, UK; and* [4]*Department of Polymer Chemistry, University of Trondheim, Norway.*

**Summary** Improvements to the original procedure of using a panel of monoclonal antibodies and magnetic microspheres for the depletion of tumour cells from bone marrow are described. These include a completely disposable system for the magnetic depletion of tumour cells coated with magnetic microspheres. Properties of a new series of microspheres are compared with the old M330 beads in their ability to deplete neuroblasts from both model systems and 50 bone marrows harvested from Stage IV neuroblastoma patients. Using human neuroblastoma cell lines labelled with the DNA intercalating, Hoechst dye 33342 a 5% tumour contamination can routinely be removed from $5\times10^6$–$5\times10^7$ nucleated cells. Analysis of the 50 purged marrows revealed that 10 were visibly contaminated with tumour (by conventional cytology and immunological procedures). In all but one case, tumour cells were removed. In this instance the tumour:bead ratio fell to 1:4 indicating the importance of maintaining a sufficient number of beads in the system. Red cell contamination of marrow was also kept extremely low so preventing possible physical blockade of bead:tumour cell interaction. Marrow engraftment was rapid in this group, apart from patients who had been exposed to high doses of alkylating agents prior to autografting.

A variety of techniques have been developed to deplete tumour cells from bone marrow harvested for autologous transplantation (Treleaven *et al.*, 1985). Of these, four have been used either experimentally or clinically for the removal of neuroblasts from marrow taken from children with Evans stage IV neuroblastoma (Evans *et al.*, 1971). Reisner (1983) has shown that, in model systems, he could obtain a limited depletion of neuroblasts from bone marrow using soybean agglutinin. Saarinen *et al.* (1985) have described, in model systems, the use of a murine monoclonal antibody of the IgG3 isotype (recognising the ganglioside GD2) and human complement for killing tumour cells in bone marrow. However the use of a single antibody and complement for purging marrow is unlikely to lead to maximal tumour kill as Gee *et al.* (1986) have demonstrated that this leads to the selection of cells of low antigen density, resistant to complement mediated cytolysis.

The derivative of cyclophosphamide, Asta Z, has been used to 'chemically purge' neuroblasts from bone marrow harvested for autologous transplantation (Douay, 1985). Whilst this is effective in model systems using relatively rapidly dividing cell lines, it may not efficiently kill fresh neuroblastoma cells, many of which may be in the G1/G0 phase of the cell cycle (Danon *et al.*, 1980). Alternatives for the chemical purging of neuroblasts from bone marrow are dopamine and ascorbic acid (Reynolds *et al.*, 1982) and the analogue of epinephrine/norepinephrine, meta-iodobenzylguanidine radiolabelled with 131-I (Buck *et al.*, 1985). In addition the photoactivation of merocyanine 540 which is reported to be selectively taken up into the membranes of certain leukaemic and neuroectodermal cell lines is another approach to investigating tumour cell kill in bone marrow (Sieber *et al.*, 1986).

The physical removal of neuroblastoma cells from bone marrow using an elutriation centrifuge has been described by Figdor *et al.*, (1985). This was reported capable of depleting only ~90% of tumour cells from marrow. An alternative physical procedure is to use a panel of monoclonal antibodies and magnetic microspheres (Treleaven *et al.*, 1984). This is effective for the removal of neuroblastoma cell lines and fresh neuroblasts from bone marrow with an efficiency of at least three logarithmic units of tumour depletion (Treleaven *et al.*, 1984). An indirect approach to targetting microspheres to tumour cells has been adopted. Initially monoclonal antibodies are added to contaminated bone marrow and these bind selectively to the tumour cells. Microspheres coated

Correspondence: J.T. Kemshead.
Received 14 May, 1986.

© The Macmillan Press Ltd., 1986

with anti-mouse Ig are then added to the system which bind to the antibody-coated cells. (Treleaven *et al.*, 1984).

Over the last two years the original method for the depletion of neuroblastoma cells from bone marrow has been considerably modified. Here we detail these modifications and report on the first 50 marrows from neuroblastoma patients treated by this technique. This represents ~30% of the total 'magnetic' marrow purges that have been undertaken for neuroblastoma and other malignancies in the collaborative groups investigating this technique.

### Materials and methods

*Preparation of microspheres*

Both the M330 and 450 series of microspheres are magnetic polymer beads of uniform size prepared by the method of Ugelstad *et al.* (1984). To obtain a monodispersed preparation of microspheres, suspensions of M330 microspheres were sonicated as described previously (Treleaven *et al.*, 1984). Preparations containing individually separate beads were prepared by simply shaking the M450 microspheres in PBS. Both series of microspheres were washed 3 times in alcohol for sterilisation and incubated overnight with sheep anti-mouse immunoglobulin (Ig) in PBS (pH 7.7) for 18 h at 4°C. The microspheres were washed using PBS containing 10% v/v human plasma protein (PPF) (4.5% albumin) directly before use to remove unbound anti-mouse Ig.

*Collection of bone marrow and preparation of a buffy coat fraction.*

Bone marrow was harvested from either/both iliac crest(s) under general anaesthetic and transferred to culture medium containing preservative-free heparin (final concentration 4 U ml$^{-1}$). Buffy coat fractions were prepared using either a IBM (Cobe) 221 or the Erytrenn cell separator (Biotest). Care was taken to reduce the red cell contamination of the buffy coat fraction to a minimum, without excessive loss of nucleated cells. If this was not achieved during the initial separation a further buffy coat was prepared when excess antibody was washed from the bone marrow (see **Results**).

*Monoclonal anitbodies and incubation of bone marrow with anti-mouse Ig coated microspheres*

A panel of purified monoclonal antibodies, selectively binding to neuroblastoma, were added to the buffy coat fraction of bone marrow as described previously (Treleaven *et al.*, 1984). Following incubation for 30 min at 4°C, the marrow was washed free of excess antibody and transferred to a 600 ml transfer bag (Travenol Lab., U.K.). M330 microspheres (100 mg) or a minimum of 150 mg of M450 microspheres were added to the marrow, and the bag gently rotated for 30 min at 4°C. Sufficient volume of RPMI 1640 tissue culture medium containing 10% PPF was added to the marrow to give a packed volume of cells no greater than 10%.

*Magnetic cell separation.*

The system currently in use is an adaptation of the original flow system, now employing disposable magnetic separation chambers. These are made from gambrohemofreeze bags. A heat sealer was used to reduce the volume of the bags to ~30 ml. Two of these modified 'chambers' were linked using a disposable Travenol 2243 connector. Fourteen samarium cobalt magnets were placed under the chambers as illustrated in Figure 1. The treated marrow was attached to the first chamber using a Travenol 2243 line. The marrow was 'pulled' through the system at a constant rate of 2.5 ml min$^{-1}$ using a peristaltic pump. This was placed after the magnetic separation chambers to avoid disruption of the tumour cell/bead complexes. The separation chambers and marrow were kept at 4°C during the purging procedure. A Travenol 2244 connector was used to link the last chamber to the pump and a similar connector used to attach a Travenol 600 ml transfer pack to the apparatus to collect marrow purged of tumour. The chambers were flushed free of haemopoietic progenitors using saline containing 10% PPF v/v to maximise cell yields.

*Tumour cell depletion and assessment of haemopoietic progenitor recovery*

In the model system studied neuroblastoma cells were initially incubated with the DNA binding dye Hoechst 33342 according to the method of Reynolds *et al.* (1985). These were titrated into either peripheral blood or bone marrow at different percentages (1–10%). Removal of tumour cells from the system was determined by estimating the number of fluorescent cells remaining in the cell mixture under UV fluorescent microscopy. Before analysis samples were mixed with trypan blue to exclude the possibility of dye being taken up by dead cells.

Tumour cell removal from fresh bone marrow was determined by indirect immunofluorescence using monoclonal antibodies and fluorescein conjugated sheep anti-mouse Ig. Several antibodies

**Figure 1** The flow system incorporating disposable chambers and samarium cobalt magnets for the depletion of tumour cells from bone marrow.

used in this study [PI153/3 (Kennett & Gilbert, 1979), A2B5 (Eisenbarth *et al.*, 1979), UJ167.11, UJ223.8] were not employed in the magnetic depletion procedure. Assays for haemopoietic progenitor cells, (CFUc, BFUe and CFUgem) were undertaken according to the method of Fausner and Messner (1979). Samples were also checked for bacterial and fungal contamination in the Bacteriology Dept., Hospital for Sick Children.

### Results

### *Magnetic microspheres*

The properties of the new M450 microspheres are listed in Table I. Unlike the M330 series of microspheres the M450 beads become completely monodipersed upon suspension in either water or PBS (no sonication needed). They are resistant to most organic solvents enabling them to be rapidly sterilised. Like the 330 series of beads which they replace, they are uniform, both in size and in the amount of magnetite they contain (Figure 2A). The major functional differences in the M450 microspheres over the old beads are a 5-fold reduction in the number of beads/mg, and a 30-fold reduction in their surface area (Table I). This reduces considerably the amount of protein needed to saturate protein binding sites on the microspheres. Only $6\,mg\,Ig\,g^{-1}$ beads will result in maximal protein binding, 15 times less than that used with the M330 series. However due to the reduction of the number of beads $mg^{-1}$ (Table I) to maintain a

**Table I** Properties of the microspheres used for tumour cell depletion.

| | *Original Microspheres (M330)* | *New microspheres (M450)* |
|---|---|---|
| Size | 3 $\mu m$ | 4.5 $\mu m$ |
| Particles (mg) | $7.9 \times 10^7$ | $1.4 \times 10^7$ |
| Surface area | $100$–$150\,m\,g^{-1}$ | $3$–$5\,m\,g^{-1}$ |
| Monodispersal without sonication | No | Yes |
| Ig binding for depletion studies | $100\,mg\,Ig\,g^{-1}$ microspheres | $<10\,mg\,Ig\,g^{-1}$ microspheres |
| Stability of binding over 7 days | >95% | >95% |
| Ability to covalently link antibody | No | Yes |

**Figure 2** A. Scanning electron micrograph of the M450 series of microspheres illustrating uniformity and monodispersal. Magnification ×8000. B. Scanning electron micrograph of microspheres binding to neuroblastoma cells. Magnification ×2000.

bead:tumour ratio of ~30:1 increased quantities of beads need to be used in the purging procedure (see below). An overall saving of 70–80% of anti-mouse Ig can however be made using the M450 rather than the M330 microspheres.

*Preparation of buffy coat fractions from bone marrow*

Although it was aimed to harvest $3 \times 10^8$ nucleated cells $kg^{-1}$ body wt from each patient, considerable variations in cell counts were obtained ($1.1$–$8.0 \times 10^8\,kg^{-1}$; $n = 50$). Volumes of harvested marrow ranged from 150–850 ml. For volumes <400 ml the Erytrenn cell separator was used in preference to the IBM apparatus for buffy coat preparation. The relatively large capacity of the IBM 2991 bag (650 ml) makes this difficult to use with small volumes of bone marrow without adding a dense inert fluid, e.g. glycerol, to reduce the internal capacity of the bag. Red cell contamination

**Figure 3** A comparison of the recovery of nucleated WBC after depleting bone marrow of tumour cells using either M330 or M450 microspheres. ▧ buffy coat fraction; □ after tumour depletion.

was kept as low as possible and calculated to be no more than 50–80 RBC/nucleated WBC. Recovery of nucleated cells after preparation of the buffy coat was found to be ~64% (range 40–130%; $n = 50$).

*Haemopoietic cell recoveries*

Figure 3 shows that purging marrow of neuroblastoma cells with either the M330 series of microspheres ($n = 14$) or the M450 beads ($n = 36$) results in similar losses of cells. In both instances a mean of 65–70% of nucleated cells were recovered, although considerable variation in the percentage recoveries were noted (M330 beads, 42–111%; M450 series, 38–87%). Recovery of cells was independent of the original cell count and although several marrows were contaminated with tumour cells these could not account for the overall losses observed.

Studies on colony assays pre and post separation with antibodies and either series of microspheres showed a small but non-selective loss of haemopoietic progenitors per $10^5$ mononuclear cells (Table II). No significant difference was noted in the losses observed using either M330 or M450 microspheres. Whilst the number of marrows purged with the magnetic bead technique was 50 in total, colony assay data were only available in full for 33 due to sample losses, infections and assay failures.

*Depletion efficiency*

Using model experiments to determine the efficiency of detecting Hoechst labelled neuro-

**Table II** Comparison of bone marrow harvested for patients with neuroblastoma treated with a panel of monoclonal antibodies and either M330 or M450 microspheres.

| | *CFUc × $10^5$ mononuclear cells* | | *BFUe × $10^5$ mononuclear cells* | | *CFUgem × $10^5$ mononuclear cells* | |
|---|---|---|---|---|---|---|
| | *Mean* | *Range* | *Mean* | *Range* | *Mean* | *Range* |
| *Original microspheres M330* | | | | | | |
| Buffy fraction | 110 | (27–211) | 33.5 | (2–81) | 5 | (0–22) |
| Post treatment | 93.5 | (2–208) | 22.5 | (0–56) | 5 | (0–26) |
| % recovery | 84 | | 67 | 76 | | |
| *n* | 13 | | 13 | | 12 | |
| *New microspheres M450* | | | | | | |
| Buffy fraction | 150 | (43–343) | 19.6 | (6–40) | 3.6 | (0–20) |
| Post treatment | 126 | (27–354) | 17.8 | (1–50) | 2.8 | (0–6) |
| % recovery | 84 | | 90.8 | | 77 | |
| *n* | 27 | | 24 | | 21 | |

blastoma cells titrated into peripheral blood mononuclear cells or bone marrow we routinely identified one malignant cell in 10,000 normal nucleated progenitors. In depletion experiments 1, 5, 10, 20% Hoechst labelled cells were titrated into between $5 \times 10^6$ and $5 \times 10^4$ normal cells. One mg of either M330 or M450 microspheres was used to deplete tumour cells from the population after incubation with anti-neuroblastoma monoclonal antibodies. Microscopic examination of multiple fields (>20,000 cells) did not identify residual neuroblasts in samples containing 1 or 5% of tumour cells (Figure 2.B). This was found to be the case for both the M330 and 450 microspheres. In these experiments as the tumour burden in the marrow was increased the number of beads added was kept constant. Therefore the ratio of beads to tumour cells fell 20 times during the experiments. Occasional fluorescent tumour cells were identified in the marrow/blood, mononuclear cells originally contaminated with 10% and 20% neuroblasts. Here, using the M450 microspheres the bead:tumour ratio became as low as 3:1. No attempts were made to increase the efficiency of the depletion technique at these levels of tumour contamination as it was not felt to be clinically relevant.

Of the 50 marrow purges reviewed 10 were undertaken on marrows that were contaminated with tumour cells (as identified by conventional cytology and immunohistology). Tumour cell contamination ranged from approximately 1–7% (Table III). In all of these cases except one, tumour cells were removed from the marrow after incubation with monoclonal antibodies and magnetic microspheres. In this instance the ratio of beads to tumour cells was ~4:1. This occurred as the marrow was not tested for the degree of tumour cell involvement prior to the purging procedure.

Whilst efficient tumour purging occurred with M450 beads at bead:tumour ratios of ~12–17:1,

**Table III** Removal of neuroblastoma cells from bone marrow using a panel of monoclonal antibodies and two different types of magnetic microspheres.

| *Beads* | *Estimated % tumour cells in buffy coat* | *Ratio beads: tumour cells in buffy coat* | *Cells detected[a] after purge* |
|---|---|---|---|
| M330 | 2 | 131:1 | None |
| M330 | 2 | 92:1 | None |
| M330 | 1 | 63:1 | None |
| M330 | 1 | 114:1 | None |
| M450 | 2 | 24:1 | None |
| M450 | 2 | 17:1 | None |
| M450 | 2.5 | 12:1 | None |
| M450 | 7 | 4:1 | 2% |
| M450 | 1.5 | 46:1 | None |
| M450 | 1 | 38:1 | None |

the errors involved in estimating the level of malignant cell contamination in marrow are high. This is due to the tumour clumps that are invariably found in the marrow. Currently we allow 30 beads/tumour cell as a minimum and this is increaced to >50:1 if red cell contamination cannot be reduced to the levels illustrated above.

*Reconstitution times*

Of the 50 marrows purged, 10 patients have been lost to follow up, many being children treated outside the U.K. Six were marrows harvested from children receiving high dose targeted radiation therapy, and it was not necessary to use this for autologous rescue (Kemshead *et al.*, 1985). Two patients relapsed between the marrow harvest and the ablative therapy and treatment was not continued. Ten marrows remain frozen to be used at a later date in the childrens' management. Data are available on 21 individuals of which 4 succumbed to infection prior to regraftment (within 30 days of giving the graft). This was not due to contamination of the marrow during the purging procedure as assessed by bacterial/fungal screening.

Thirteen of 17 patients were given a high dose ablative protocol in first remission. Of these 6 marrows were purged using the M330 beads. The mean time (T) for recovery to 500 neutrophils was 21.5 days (range 17–24) and 50,000 platelets 30.3 (range 19–43) days. The remaining 7 were purged using the M450 microspheres and similar recovery times were noted T-500 neutrophils, 22 days (range 14–36) and T 50,000 platelets, 33 days (range 19–38). The remaining 4 patients who were grafted in second remission, had considerably longer recovery times (mean T500 neutrophils 46 days; mean T50,000 platelets 81.5 days). All of these had been previously exposed to relatively high doses of alkylating agents, which presumably caused damage to either marrow progenitors or stromal elements associated with haemopoietic recovery.

It is not possible to acquire accurate data on the survival of this group of patients as they have been treated in different centres, using different induction and ablative protocols (see **Discussion**).

### Discussion

The use of magnetic microspheres and panels of monoclonal antibodies for the depletion of tumour cells from bone marrow is an extremely fast, efficient and cost effective procedure (Treleaven *et al.*, 1984). The modifications described here represent considerable improvements over the original method, economising on reagents, the time involved in the procedure, and allowing a completely disposable system to be used for the magnetic separation chambers. The indirect approach to depleting tumour cells from bone marrow allows for the saturation of antigen binding sites on malignant cells, and attempts to overcome the problem of antigen heterogeneity known to occur in tumour cells (Kemshead *et al.*, 1983).

Adaptations of the indirect procedure described above are currently being explored by others in experimental systems. Kvalheim *et al.* (personal communication) is using high affinity IgM antibodies directly conjugated to M450 microspheres to deplete lymphoma cells from bone marrow and Gee *et al.* (personal communication) used anti-T and anti-neuroblastoma antibodies pre-complexed with anti-mouse Ig coated microspheres to remove T cells and neuroblasts from peripheral blood and bone marrow respectively. Currently we are exploring the use of magnetic microspheres coated with material other than anti-mouse Ig. Beads coated with C1q, a component of complement, can recognise antibody only when complexed with antigen suggesting that these may be added to bone marrow contaminated with tumour at the same time as the panel of monoclonal antibodies (Sawada *et al.*, 1986).

The indirect system of depleting tumour cells from bone marrow has been used to purge marrow harvested from 14 children with acute lymphoblastic leukaemia. Obviously in these cases a different panel of antibodies has been applied to the marrow. Whilst the reagents show very low cross reactivity with cells in normal bone marrow (<1%), the levels of nucleated cell and committed haemopoietic cell progenitors recovered are considerably and consistently lower than for the neuroblastoma patients (Kemshead, in preparation). These patients have engrafted well on the whole, but the observation suggests that the use of different antibodies with the magnetic microspheres may considerably alter cell recoveries obtained and by implication the efficiency of tumour cell removal.

No direct comparison of other purging procedures has been undertaken for neuroblastoma on marrows used clinically, since in the laboratory other procedures have produced results that are inferior to the magnetic separation technique. However, Favrot *et al.* (1985) have compared the efficiency of antibody and complement and magnetic tumour cell depletion using marrow contaminated with Burkitt's lymphoma cell lines. Using a modified system to that described above employing a mononuclear cell fraction rather than buffy coat and double depletion procedure, Favrot and colleagues report a depletion efficiency of 4 logarithmic units which is equal to that achieved with antibody and complement.

Whether buffy coat preparations are preferable to mononuclear cell preparations for immunomagnetic depletion remains controversial. Mononuclear cell preparations have the advantage that the levels of contaminating RBC are very low which thus avoids physical inhibition of the binding of microspheres to tumour cells. However cell losses are high and the added complexity and time involved in the preparation of a mononuclear cell fraction suggests that, if sufficient red cell depletion of a buffy coat fraction can be achieved, this would be the method of choice. At red cell levels of 50–80 cells/nucleated WBC and approximately the same ratio of microspheres: tumour cells, no inhibition of beads binding to neuroblasts was noted.

The M450 microspheres offer considerable advantage over the old M330 beads and those produced by other laboratories. These beads have hydroxyl groups on their surface allowing monoclonal antibodies, anti-mouse Ig or other antibody binding molecules to be covalently linked to the surface of the microspheres. This should further standardise the depletion technique used for neuroblastoma in different laboratories.

The clinical need to purge bone marrow in Stage IV neuroblastoma needs further evaluation. An analysis of the group of patients treated in association with this laboratory is difficult due to the different induction protocols employed and the different ablative regimens used in association with the autograft. Preliminary results from our studies and those of T. Philip (using standard induction and ablative regimens) suggest that the role of high dose chemo/radiotherapy in children with Stage IV neuroblastoma is one of consolidation therapy for children either in complete remission or having minimal residual disease following conventional combination chemotherapy. Where children are in overt relapse or partial remission with a considerable tumour burden the high dose 'megatherapy' given appears insufficient to ablate the tumour *in vivo*. In these cases where relapse/progression often occurs post megatherapy and transplantation, active disease is almost invariably found at sites of original disease although marrow involvement is also often observed.

Although it is too early to be confident of survival, several children given high dose radio/chemotherapy when in complete remission remain well over two years post transplantation. The role of purging in this group is difficult to establish. The known patchy involvement of neuroblastoma in bone marrow makes accurate detection of tumour extremely difficult. In two cases of the 50 described here, marrow aspirates appeared free of tumour cells one month prior to the harvest for transplantation and yet a heavy infiltrate was found when the autograft was prepared. These observations provide the most compelling reasons for purging marrow from Stage IV neuroblastoma patients apparently in complete remission. The current ablative regimens used in Stage IV neuroblastoma carry a considerable risk of toxicity. As well as facilitating the clinical application of this type of therapy, research efforts should continue to improve the purging procedure and reduce overall toxicity. This may be accomplished in the future by the further *in vivo* use of monoclonal antibodies as agents to target radioisotopes to minimal residual disease. Toxicity of this type of therapy is currently being assessed but it is likely to be considerably less than the problem associated with giving external beam total body irradiation to children.

This work was partially funded by the Imperial Cancer Research Fund. We are grateful to SINTEF, Norway, for supplying the magnetic microspheres for the purging procedures. In addition we thank the Oncology centres in the UK, Europe, USA and Australia for co-operating in these investigations. We are also indebted to the air crew, customs officials and the traffic police in assisting in the rapid transit of bone marrow to the laboratory. Finally we acknowledge the Haematology Dept. in the Hospital for Sick Children for the use of equipment and S. Watts for typing this manuscript.

### References

BUCK, J., BRUCHELT, G., GIRGERT, R., TREUNER, J. & NIETHAMMER, D. (1985). Specific uptake of 125-I meta-iodobenzylguanidine in the human cell line SK-N-SH. *Cancer Res.*, **45**, 6366.

DANON, Y.L., EPSTEIN, M.B., SIEGEL, M.M. & 4 others (1980). Flow microfluorometric analysis of human neuroblastoma DNA distributions. In: *Advances in Neuroblastoma Research*, Evans, A.E. (ed.), p. 287. Alan R. Liss, New York.

DOUAY, L. (1985). Cyclophosphamide derivatives for *in vitro* cleansing of leukaemic bone marrow. In *Recent Advances in Autologous Bone Marrow Transplantation in Onco-Haematology*, Herve P. & Gorin N.C. (eds.), p. 427. Librairie Arnette, Paris.

EISENBARTH, G.S. WALSH, F.S. & NIRENBERG, M. (1979). Monoclonal antibody to a plasma membrane antigen of neurons. *Proc. Natl Acad. Sci. USA*, **76**, 4913.

EVANS, A.E., D'ANGIO, G. & RANDOLPH, J. (1971). A proposed staging system for children with neuroblastoma. *Cancer*, **27**, 374.

FAUSNER, A.A. & MESSNER, H.A. (1979). Identification of megakaryocytes, macrophages and eosinophils in colonies of human bone marrow containing neutrophilic granulocytes and erythroblasts. *Blood*, **53**, 1023.

FAVROT, M.C., PHILIP, I., MARITAZ, O., GARCON, N. & PHILIP, T. (1985). Immunological bone marrow purging procedure in Burkitt's lymphoma. Evaluation by a liquid cell culture assay. In *Recent Advances in Autologous Bone Marrow Transplantation in Onco-Haematology*, Herve P., & Gorin, N.C. (eds.), p. 455. Librairie Arnette, Paris.

FIGDOR, C., VOUTE, P.A., DE KRAKER, J., BONT, W. & VERNIE, L. (1985). Physical separation of neuroblastoma cells from bone marrow. In *Advances in Neuroblastoma Research*, Evans, A.E. (ed.), p. 459. Alan R. Liss, New York.

KEMSHEAD, J.T., TRELEAVEN, J.G., GIBSON, F.M., UGELSTAD, J., REMBAUM, A. & PHILIP, T. (1985). Monoclonal antibodies and magnetic microspheres used for the depletion of malignant cells from bone marrow. In *Advances in Neuroblastoma Research.* Evans, A.E. *et al.* (eds.), p. 413. Alan R. Liss, New York.

KEMSHEAD, J.T., GOLDMAN, A., FRITSCHY, J., MALPAS, J. & PRITCHARD, J. (1983). Use of panels of monoclonal antibodies in the differential diagnosis of neuroblastoma and lymphoblastic disorders. *Lancet*, **i**, 13.

KENNETT, R.H. & GILBERT, F. (1979). Hybrid myelomas producing antibodies against a human neuroblastoma antigen present on foetal brain. *Science*, **203**, 1120.

REISNER, Y. (1983). Differential agglutination by soybean agglutinin of human leukaemia and neuroblastoma cell lines: Potential application to autologous bone marrow transplantation. *Proc. Natl Acad. Sci.*, **80**, 6657.

REYNOLDS, C.P., REYNOLDS, D.A., FRANHEL, E.P. & SMITH, R.G. (1982). Selective toxicity of 6-OH dopamine and ascorbate for human neuroblastoma *in vitro*: A model for clearing marrow prior to ABMT. *Cancer Res.*, **42**, 1331.

REYNOLDS, C.P., MOSS, T.J., SEEGER, R.D., BLACK, A.T. & WOODY, J.N. (1985). Sensitive detection of neuroblastoma cells in bone marrow for monitoring the efficacy of marrow purging procedures. In *Advances in Neuroblastoma Research*, Evans, A.E. *et al.* (eds.), p. 425. Alan R. Liss, New York.

SAARINEN, U.M., COCCIA, P.F., GERSON, S.L., PELLEY, R. & CHEUNG, N.V. (1985). Eradication of neuroblastoma cells *in vitro* by monoclonal antibody and human complement: Method for purging autologous bone marrow. *Cancer Res.*, **45**, 5969.

SAWADA, T., HEATH, L., RICHMOND, F., UGELSTAD, J., LIBERTI, P. & KEMSHEAD, J. (1986). Magnetic microspheres coated with the complement component C1q for the removal of tumour cells from bone marrow. UCLA Symposia on Molecular and Cellular Biology. (In press.)

SIEBER, F., & SIEBER-BLUM, M. (1986). Dye mediated photosensitisation of murine neuroblastoma cells. *Cancer Res.* (In press.)

TRELEAVEN, J.G. & KEMSHEAD, J.T. (1985). Removal of tumour cells from bone marrow: An evaluation of the available techniques. *Hematol. Oncol.*, **3**, 65.

TRELEAVEN, J.G., GIBSON, F.M., UGELSTAD, J. & 4 others (1984). Removal of neuroblastoma cells from bone marrow with monoclonal antibodies conjugated to magnetic microspheres. *Lancet*, **i**, 70.

UGELSTAD, J., REMBAUM, A., KEMSHEAD, J.T., NUSTAD, K., FUNDERUD, S. & SCHMID, R. (1984). Preparation and biomedical applications of monodiperse polymer particles. In *Microspheres and Drug Therapy: Pharmaceutical Immunological and Medical Aspects*, Davies, S.S. *et al.* (eds.), Elsevier Science Publishers, B.V.